# Psychological Distress and Adolescents’ Cyberbullying under Floods and the COVID-19 Pandemic: Parent–Child Relationships and Negotiable Fate as Moderators

**DOI:** 10.3390/ijerph182312279

**Published:** 2021-11-23

**Authors:** Yuchi Zhang, Chengpei Xu, Hanyue Dai, Xiaoyu Jia

**Affiliations:** 1 Department of Educational Technology, School of Smart Education, Jiangsu Normal University, Xuzhou 221116, China; psydreamzhang@sina.cn (Y.Z.); 3020190674@jsnu.edu.cn (C.X.); 3020190651@jsnu.edu.cn (H.D.); 2 Jiangsu Engineering Research Center of Educational Informatization, Jiangsu Normal University, Xuzhou 221116, China; 3 Center for Studies of Education and Psychology of Ethnic Minorities in Southwest China, Southwest University, Chongqing 400700, China

**Keywords:** cyberbullying, psychological distress, parent–child relationships, negotiable fate, COVID-19, culture contexts

## Abstract

Since the outbreak of coronavirus disease 2019 (COVID-19), adolescents in 70 countries have suffered the COVID-19 pandemic and flood disasters simultaneously. Although antecedent cyberbullying variables have attracted significant research attention, the effects of psychological distress and the potential mechanisms of cyberbullying among adolescents under multiple disasters remains unclear. Based on social-ecological system theory, this study examines the moderating effects of parent–child relationships and the negotiable fate on the relationship between psychological distress and cyberbullying. A total of 1204 middle school students (52.4% boys) who suffered from floods and the COVID-19 pandemic from Zhengzhou City, China, are the participants. The results reveal that psychological distress was positively related to adolescent cyberbullying during a disaster. Parent–child relationships and negotiable fate significantly moderate the relationship between psychological distress and cyberbullying. Specifically, high parent–child relationships and a high negotiable fate could protect adolescents from the negative effects of psychological distress of cyberbullying. For adolescents with low or high parent–child relationships and low negotiable fate, the links between psychological distress and cyberbullying are stronger. These findings underline the significance of considering the interaction of psychological distress, parent–child relationships, and negotiable fate when examining adolescents’ cyberbullying during disasters.

## 1. Introduction

Since the beginning of 2020, adolescents have suffered the global coronavirus disease 2019 (COVID-19) pandemic. Unfortunately, people in 70 countries simultaneously suffered flood disasters at the same time [1]. For example, at the end of July 2021, an extraordinary rainstorm battered Henan province, China, and caused floods in Zhengzhou City, the provincial capital city of Henan province. The amount of rainfall in Zhengzhou over three days was equal to the average annual amount [2]. By 3 August 2021, floods killed 302 people in Zhengzhou and affected nearly 14.53 million people [2]. Moreover, the new wave of the coronavirus disease 2019 (COVID-19) pandemic attacked Zhengzhou City following the floods and rapidly affected 63 people within four days [3]; the local government closed some districts, restricting people from stopping the spread of the outbreak [3]. The flood and the COVID-19 pandemic became a “double disaster” for Zhengzhou adolescents, which may have caused adolescents’ psychological symptoms and distress [4,5]. Several studies have revealed that a single type of natural disaster, or the COVID-19 pandemic, can cause psychological distress in adolescents, such as depression, anxiety, and stress [6,7,8,9]. The prevalence of psychological distress among adolescents ranges from 10% to 30% in Western and Eastern societies [10]. In addition to mental health, limited studies have explored the effects of psychological distress on adolescents’ cyber problem behaviors, such as cyberbullying during the COVID-19 pandemic. Cyberbullying is a complex and serious problem in the digital age worldwide [11,12]; it refers to “Any behavior performed through electronic or digital media by individuals or groups that repeatedly communicates hostile or aggressive messages intended to inflict harm or discomfort on others” [13]. Cyberbullying has severe consequences on adolescents’ mental health, and adolescents with COVID-19 may have more free time to use the Internet, which facilitates possible cyberbullying. Thus, urgent research and interventions are required [13]. 

Previous studies have been limited in several ways. First, to the best of our knowledge, no empirical study has explored the relationship between psychological distress and adolescents’ cyberbullying behaviors in the context of double disasters simultaneously (i.e., floods and the COVID-19 pandemic in the current study) [14,15,16]. Considering that double disasters are a common phenomenon in human societies, it is urgent to explore these issues. Based on general strain theory, the current study adds to the literature by assuming that the double disaster would cause adolescent strain (psychological distress level), and a higher level of psychological distress would trigger more cyberbullying behaviors in order to buffer their strain during a disaster [14,15]. Thus, our first research question is to test the relationship between adolescents’ psychological distress and cyberbullying in double disasters.

Second, no existing study has examined the potential mechanisms of psychological distress in cyberbullying, let alone under diverse cultural contexts (e.g., non-Western culture). According to social-ecological theory, adolescents’ development is the consequence of the interaction between different ecological system-level factors, such as microsystems (parent–child relationships, PCRs) and macrosystem (culture belief) factors [17]. These gaps in the literature hinder our understanding of cyberbullying behaviors during disasters. High-quality PCR (microsystem level factor) is considered to play a significant role in adolescents’ mental health and problem behaviors [18]. Despite the notable findings, previous studies have not specifically contended whether high-quality PCRs can buffer adolescents’ perceived psychological strain and cushion the negative effects of psychological distress on cyberbullying behaviors in disasters [19,20]. Accordingly, our second research question is to explore the moderating effects of PCRs on the direct links between psychological distress and cyberbullying in double disasters.

Moreover, based on social-ecological theory, macrosystem factors may moderate the conditions or process of microsystems (PCRs in the current study), which implies that the macrosystem may further moderate the moderating effects of PCRs. However, none of the studies have fully explored the complex interactions between microsystems and macrosystem factors on direct links. Negotiable fate (macrosystem factor) is a dominating cultural belief about fate, which is more general in Eastern cultures [21,22]. Our third research question is to explore the moderating effects of negotiable fate and PCRs on the relationship between psychological distress and cyberbullying in double disasters.

Taken together, the present study explores whether negotiable fate could further moderate the moderation effects of PCRs on the associations between psychological distress and cyberbullying behaviors among Chinese adolescents who suffer from floods and the COVID-19 pandemic in Zhengzhou City (three-way interaction effects model) through a cross-sectional design.

### 1.1. Psychological Distress and Cyberbullying in Disasters

Cyberbullying is a worldwide social problem in the digital age [11], leading to cyber victimizations’ mental health problems [23]. Large-scale studies have revealed that the prevalence of cyberbullying among adolescents in the United States was 22.7% [12] and 14% to 57% in mainland China [24]. With the long existence of COVID-19, several empirical studies have explored the antecedent variables of cyberbullying in COVID-19 pandemic contexts. However, the majority of studies adopt adult participants [14,15,16]. The predictive factors of adolescent cyberbullying behavior remain unclear. To our knowledge, no study has tested adolescents’ cyberbullying perpetration in the context of two types of disasters (i.e., flood: natural disaster, COVID-19: pandemic disaster). For the design of evidence-based cyberbullying prevention programs in the COVID-19 era, it is necessary to explore this issue.

Disasters increase the risk of psychological distress for adolescents [4,5]. Research has found that floods and COVID-19 could lead to higher levels of depression, anxiety, and stress among adolescents [4,25,26]. The present study uses the term “psychological distress” to show the adolescents’ level of depression, anxiety, and stress distress [27]. According to the general strain theory, stress from a disaster could be a strain to develop an individual’s negative emotions. Consequently, individuals exhibit deviant behaviors to mitigate their feelings [14,28,29]. Because of the multiple disasters, adolescents in Zhengzhou City have a higher risk of experiencing higher levels of psychological distress, making them angry or frustrated. Consequently, adolescents may choose cyberbullying behaviors to mitigate these undesirable feelings. One study indicated that adults’ personal (e.g., being affected by COVID-19) and proximal (e.g., knowing people affected by COVID-19) experiences with COVID-19 were positively related to adults’ cyberbullying behaviors [14]. A study examining 10–17-year-old students revealed that an individual’s stress or strain leads to cyberbullying behaviors [29]. Therefore, the present study assumes the following:

**Hypothesis** **1.** *Adolescents’ psychological distress is positively related to their cyberbullying behaviors in the context of disasters.*

### 1.2. Moderating Effects of Parent–Child Relationships

Social-ecological system theory claims that children’s development is a consequence of interactions between different system-level factors. PCRs, reflecting the conflict and closeness between parents and their children, play an important role in adolescents’ development [30], ([31], pp. 204–222). As the stress-buffering model posits, positive social support from interpersonal relationships buffers the damage of stress on children’s negative mental or behavioral outcomes [19,20]. In the COVID-19 pandemic and flood breakdown time, parents commonly interacted with adolescents during home confinement [32]. Previous research found that high quality PCRs moderated the relationship between parents’ and children’s emotional health during COVID-19, and high PCR buffers cyberbullying perpetration [18,33]. Surprisingly, to our knowledge, no empirical study has examined the moderating effects of PCRs on the relationship between psychological distress and cyberbullying in double disasters. Based on the previous literature, we anticipate parents to show their children’s warmth and support throughout the flood and COVID-19 outbreak, which may buffer the negative links between psychological distress and cyberbullying behaviors. Conversely, parents have more conflict with their children, which may aggravate adolescents’ psychological distress. To mitigate the higher strain caused by disasters and their poor PCRs, adolescents would engage in more cyberbullying behaviors.

**Hypothesis** **2.** *PCRs moderate the relationship between adolescents’ psychological distress and cyberbullying behaviors during disasters. Specifically, high-quality PCRs may buffer the links between psychological distress and cyberbullying behaviors, whereas the positive relationship between psychological distress and cyberbullying would be stronger for adolescents who perceived low-quality PCRs.*

### 1.3. Moderated Moderating Effect of Negotiable Fate

Social-ecological system theory claims that the macrosystem, such as cultural beliefs (i.e., negotiable fate in the present study), could interact with microsystem-level factors (i.e., PCRs). Specifically, the macrosystem affects certain conditions and processes within microsystems (e.g., the moderating effects of PCRs) [17,34]. Although the significant role of the macrosystem (i.e., cultural belief in the present study) is claimed by social-ecological system theory, few empirical studies have tested the role of cultural beliefs in cyberbullying, let alone its interaction effects between different system levels on cyber behaviors during double disasters. 

Negotiable fate refers to the belief that adolescents “can negotiate with fate for control, and they do this by exercising personal agency within the limits that fate has determined,” [21,22] by reflecting on individuals admitting to the authority of fate without hopelessness [22]. This concept can be outlined by a common saying: “If fate hands you lemons, make lemonade.” Individuals who are highly subscribed to negotiable fate still use positive coping to solve problems and attain their personal goals under the limitation of their fate, whereas individuals with lower negotiable fate may deny that their efforts could compensate for their fate constraints [22]. A highly negotiable fate encourages individuals to pursue their goals and engage in active coping under their fate constraints. However, little research has explored the role of negotiable fates in cyberbullying.

Based on the social-ecological system theory [17], the current study posits that the moderating effect of PCRs (microsystem-level factor) on the relationship between psychological distress (individual-level factors) and cyberbullying behaviors may differ for adolescents’ negotiable fate (macrosystem-level factor). On the one hand, adolescents with high levels of negotiable fate would acknowledge the fate limits, such as the flood- and COVID-19-induced social isolation, water and electricity outages, damage to property, and restrictions on one’s social life and studies. However, these adolescents would still believe in their efforts to help them attain their personal agency. Previous research supported this inference by revealing that both higher negotiable fate and PCRs are both closely related to active coping behaviors [22,35,36,37]. A high belief in negotiable fate is positively related to active coping strategies [22]. As previous research suggests, high PCRs help adolescents attain more warm support and coping suggestions from their parents; thus, they are more likely to develop more adaptive and active coping strategies, instead of choosing maladaptive coping behaviors when they feel stressed, anxious, or depressed [37]. Thus, adolescents with a high negotiable fate and high PCRs may use more active coping strategies to solve the emotional problem of double disasters and perform less maladaptive coping behaviors (i.e., cyberbullying). 

On the other hand, adolescents with low PCRs but high negotiable fate, according to the concept of negotiable fate, may tend to perceive worse PCRs as determined by fate, similar to the double disasters they suffered; thus, they may try to use available resources to actively cope with multiple stresses (double disasters and worse PCRs) within the limits. As a result, these adolescents would prefer to select more adaptive coping behaviors when they felt strain by psychological distress in double disasters, instead of cyberbullying perpetration. In sum, a high negotiable fate may buffer adolescents’ strain from disasters and protect them from performing less cyberbullying behavior by psychological distress, regardless of their PCRs.

Based on the social-ecological system theory, we inferred that negotiable fate as an important macrosystem factor may have a greater determining effect on adolescent development, which moderates the effect of the high or low PCRs if adolescents have a low negotiable fate [17]. On the one hand, we assume that a low negotiable fate and low quality of PCRs are risk factors for the direct links between psychological distress and cyberbullying in disasters. Kraemer et al. suggested that risk factors increase an individual’s susceptibility to stress, which leads to a higher level of psychological distress [38]. Adolescents who have worse relationships with their parents cannot attain warm support and parents’ coping suggestions or coaching, which are risk factors for adolescents’ mental states and the ability to cope in double disasters [37]. If adolescents also deny their efforts and assert their uncontrolled life [22], these beliefs may magnify the risk effects of worse PCRs and facilitate more stress susceptibility. Consequently, these adolescents would perform more cyberbullying when they perceived strain due to double disasters and worse quality of PCRs.

On the other hand, for adolescents with high parent–child relationships and low negotiable fate, the links between psychological distress and cyberbullying may also be stronger. According to the theoretical framework of negotiable fate, adolescents who have low negotiable fate believe that fate has predetermined everything for them [22]. They would not tend to mobilize all of their social resources (i.e., PCRs in our study) and did not focus on how to solve the problems of double disasters by adopting active coping strategies [21,22]. Thus, although adolescents may high PCRs, they may not adopt their parents’ coping suggestions (potential social resources) to solve the problems because they believe that nothing is in their control or that they have the ability to change in disasters. As no related evidence exists, we suggest that these adolescents may engage in more cyberbullying, because they could not adopt active coping strategies or obtain positive social support to buffer the strain caused by psychological distress in disasters.

Considering that cyberbullying is a type of maladaptive coping behavior, adolescents with high psychological distress and low negotiable fate would still exhibit more cyberbullying behaviors, regardless of their relationship with their parents.

**Hypothesis** **3a** **(H3a).** *Negotiable fate moderates the moderating effects of PCRs on the relationship between psychological distress and cyberbullying behaviors among Chinese adolescents under flood and COVID-19 disasters*.

**Hypothesis** **3b** **(H3b).** *A positive relationship between psychological distress and cyberbullying behaviors is not significant for adolescents with a high negotiable fate and high or low PCRs*.

**Hypothesis** **3c** **(H3c).** *The links between psychological distress and cyberbullying behaviors are stronger for adolescents with low negotiable fates and low or high PCRs*.

### 1.4. The Current Study

**Figure 1:**
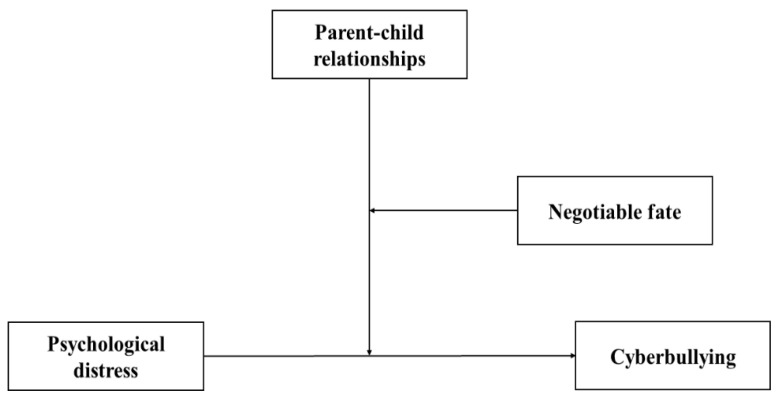
Hypothesized conceptual model.

Through merging the social-ecological system theory and general strain theory [17,28], the present study aims to deepen our understanding by exploring the mechanisms of psychological distress on cyberbullying behaviors among Chinese adolescents in Zhengzhou City, suffering from floods and COVID-19 disasters at the end of July 2021, by using a moderated moderation model (three-way interaction effects analysis). Specifically, we tested whether PCRs moderated the positive links between psychological distress and cyberbullying and whether the moderating effects of PCRs depend on the degree of negotiable fate. Figure 1 illustrates the conceptual model.

## 2. Materials and Methods

### 2.1. Participants and Procedure

A total of 1207 middle school students were recruited (52.4% boys, *n* = 633), with ages ranging from 12 to 17 years (*M* = 14.36, *SD* = 0.94). Participants were in 7th to 9th grade (7th grade: *n* = 460; 8th grade: *n* = 414; 9th grade: *n* = 333). All the participants and their parents provided informed consent. All the parents allowed their children to engage in this study, and all participants provided written informed consent online.

The online survey, performed by an online survey website (https://www.wjx.cn (accessed on 6 August 2021), was conducted from August 6th to 9th, 2021 (after approximately half a month of the Zhengzhou flood, and seven days after the COVID-19 outbreak in Zhengzhou). The teachers in the school informed the students and provided instructions on how to use the social network software. Participants only needed to agree and sign informed consent online before they could access the formal survey webpage. After completing the survey, the participants were thanked for the opportunity to conduct research through online texts.

### 2.2. Measures

#### 2.2.1. Psychological Distress

Psychological distress was measured using the 21-item Depression, Anxiety and Stress Scale (DASS-21) [39], which is widely used to measure clinical or nonclinical adolescent populations [40,41,42]. Participants rated the items on a 4-point scale (0 = did not apply to me at all, 3 = applied to me very much or most of the time) since the flood began in Zhengzhou City, China. A higher total score indicates higher psychological distress (sample item: life is meaningless). In the current sample, Cronbach’s alpha for the total score was 0.94.

#### 2.2.2. Parent–Child Relationships

PCR was performed using a four-item child-reported scale [43]. Participants rated each item on a 5-point Likert scale (1 = very untrue, 5 = very true) to report their affections between parents and themselves (sample item: My parents always treat me with love and affection). The Cronbach’s alpha for this study was 0.88.

#### 2.2.3. Negotiable Fate

Negotiable fate was measured using an adapted version of the negotiable-fate questionnaire for four items [44]. Participants rated each item on a 6-point Likert scale to indicate the extent of their agreement with each statement. The sample item of this scale is: “My efforts can compensate for my fate” [21]. The negotiable fate score was the average value of the four items. A higher score indicated that the participants held a higher belief in negotiable fate. The Cronbach’s alpha for this study was 0.88.

#### 2.2.4. Cyberbullying

Cyberbullying in flood disasters and the COVID-19 pandemic were measured using the three-item adaption version of the perpetration subscale of the cyberbullying involvement scale [45,46,47]. Participants were asked to rate their behaviors since the Zhengzhou flood in July 2021 on a 5-point Likert scale (1 = never, 2 = rarely, 3 = sometimes, 4 = once a week, 5 = daily) (sample item: “I have posted mean things about other kids on social media”). A higher score indicated a higher level of cyberbullying behavior during disasters. The Cronbach’s alpha for this study was 0.86.

#### 2.2.5. Covariate Variables

The current study used sex (male = 1, female = 2) [48], grade (7th to 9th) [49,50], and experience of being bullied as covariate variables, as these variables could affect cyberbullying [51]. The experience of being bullied was measured using the adapted version of the bullied scale [52]. Participants rated the items on a 5-point Likert scale (0 = never, 1 = 1–2 times, 2 = about once a week, 3 = 2–3 times a week, and 4 = more often) to show their bullying experience in school during the last six months.

### 2.3. Data Analysis

The moderated moderation model (three-way interaction effects) was analyzed using SPSS 23.0 and the PROCESS macro program [53]. According to our hypothesis, psychological distress is an independent variable, PCR and negotiable fate are moderating variables, and cyberbullying is an outcome variable. First, descriptive statistics, a correlation analysis, and common method bias tests on the data were tested using SPSS23.0. Second, we examined the main effects of psychological distress, the moderating effects of PCRs, and the moderated moderation model (three-way interaction effects) through Model 3 of the PROCESS macro program. Five thousand bootstrap repeated sampling tests were used to test the moderating effects of PCRs (for H1 and H2) and the moderated moderation effect (for H3a–c). If the 95% confidence interval (CI) did not include 0, the moderated moderation effect was significant [53,54].

## 3. Results

Three students responded in a patterned manner. Participants’ responses were excluded from the study. The final sample comprised of 1204 students (7th grade: *n* = 459; 8th grade: *n* = 414; 9th grade: *n* = 331).

Harman’s single-factor test was performed to examine the common method bias. The results reveal that the load of the first factor was 32.66%, indicating that there was no significant common method bias in the present study [55].

### 3.1. Preliminary Analyses

The correlations between the variables, mean, and standard deviations of the variables are presented in Table 1. Psychological distress was negatively correlated with PCRs, negotiable fate, and positively related to cyberbullying and gender (*ps* < 0.05). Both PCRs and negotiable fates were negatively related to cyberbullying (*p* < 0.01).

### 3.2. The Moderating Effect and Moderated Moderating Effect

We used Model 3 of the SPSS Hayes PROCESS macro to test the direct effect and moderation analyses [53]. As seen in Table 2, psychological distress was significantly related to cyberbullying in disasters (*β* = 0.03, *SE* = 0.01, *t* = 2.30, *p* < 0.05, 95% CI [0.005, 0.06]).

**Figure 2:**
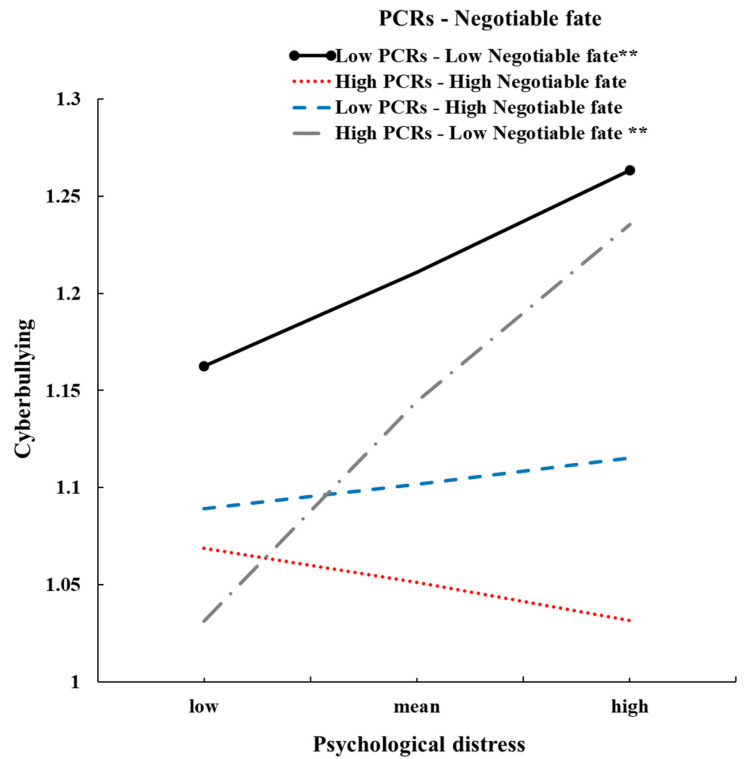
Three-way interaction effects of psychological distress by negotiable fate and parent–child relationships for adolescents’ cyberbullying under two disasters in Zhengzhou (** *p* < 0.01).

As expected (Figure 2 and Table 2), the three-way interaction of psychological distress × PCR × negotiable fate was significant (*β* = −0.02, *p* < 0.05, 95% CI [−0.03, −0.002]). However, either PCR, negotiable fate, or psychological distress could be seen as moderating variables. According to previous research procedures, and in line with our hypothesis [56,57], the present study fixed psychological distress as the independent variable and performed a simple slope to better understand the nature of the moderated moderating effect. As depicted in Figure 2, simple slope analyses indicated that the association between psychological distress and cyberbullying was non-significant when participants perceived high quality of PCRs (1 SD above the mean) and a high level of negotiable fate (1 SD above the mean) (*b_simple_* = −0.02, *t* = −0.76, *p* = 0.44, 95% CI [−0.07, 0.03]).

When the level of negotiable fate is low, high levels of cyberbullying occur when participants have higher psychological distress during disasters, regardless of the level of PCR (*ps* > 0.05).

The results indicate that the moderating effects of PCRs on the relationship between psychological distress and cyberbullying were non-significant (β = 0.002, *SE* = 0.01, 95% CI [−0.02, 0.02], *t* = 0.19, *p* = 0.85).

## 4. Discussion

Although previous studies have suggested a negative link between psychological distress and individuals’ cyberbullying behaviors [14,16], there remains a gap in understanding the role of psychological distress and its potential underlying process on cyberbullying behaviors among adolescents who suffer from COVID-19 and natural disasters in Zhengzhou City, China [58]. Research on cyberbullying underestimates the complex interactions between different ecological systems, hindering our understanding of the development of cyberbullying. Our findings demonstrate a significant moderating effect of PCRs and negotiable fate on the relationship between psychological distress and cyberbullying in disasters.

### 4.1. The Effects of Psychological Distress on Cyberbullying in Multiple Disasters

The psychological distress level positively predicted the cyberbullying behaviors of Zhengzhou adolescents. This finding supports Hypothesis 1 and is consistent with previous studies. According to the general strain theory, Zhengzhou adolescents who suffer two disasters in one month have higher strain levels, which could be reflected by their higher psychological distress [14,28]. As these strain states are undesirable, adolescents may send rude, aggressive, or nasty comments to someone online to release their higher strain states. Barlett et al. found that individuals under the breakdown of COVID-19 engaged in more cyberbullying behaviors [15]. Our findings support previous research and expand the general strain theory to cyberbullying among adolescents experiencing multiple disasters.

### 4.2. The Moderated Moderating Effects of Negotiable Fate and Parent–Child Relationships

Consistent with Hypothesis 3a, negotiable fate and PCRs moderate the relationships between psychological distress and cyberbullying behaviors among Chinese adolescents under flood and COVID-19 disasters. Specifically, high negotiable fate and high PCRs protect adolescents’ cyberbullying from their psychological distress during disasters (H3b), whereas low negotiable fate and low or high PCRs are risk factors for cyberbullying behaviors (H3c). Our findings support and extend the social-ecological system theory by highlighting the interaction with microsystems (parent–child relationships, PCRs), macrosystems (culture belief), and individual factors (psychological distress) in cyber problem behaviors in double disaster contexts [17]. Negotiable fate as a macrosystem could affect the process of PCR (microsystems) at high or low levels. These findings highlight the importance of fully exploring multiple-level factors and their interaction effects.

Specifically, our findings confirm that the combination of higher belief in negotiable fate and high PCRs reduced to stronger protective effects on the relationships between psychological distress and cyberbullying (high negotiable fate-high PCRs: *b_simple_* = −0.02; high-negotiable-fate-low PCRs: *b_simple_* = 0.01). Adolescents who have stronger beliefs about their ability to negotiate fate by taking actions to improve fated outcomes; therefore, they may actively mobilize their parents’ warmth emotional support and learn from their parents’ coping suggestions [22]. Meanwhile, they may adopt more active coping strategies during periods of flood and the pandemic. Therefore, they have less psychological distress, and they prefer to choose more adaptive coping strategies to solve emotional distress in disasters, instead of cyberbullying. Adolescents with a high negotiable fate but low levels of PCR may perceive their worse PCRs as a part of many fate constraints they could not fully overcome, just like that of double disasters. Thus, they may also set more active coping strategies to take action to improve fated outcomes [22]. As a result, they also did not prefer passive coping strategies, such as cyberbullying, because it could not help them effectively buffer their psychological distress during disasters.

Interestingly, low negotiable fate and low or high PCRs are risk factors for cyberbullying in double disasters. First, our results support Kraemer et al. ’s theoretical perspective, which argues that multiple risk factors can increase individuals’ susceptibility to stress and induce negative outcomes [38]. Low negotiable fate is a maladaptive coping strategy that prevents adolescents from focusing on problem solving and increases the risk of stress [22]. Low PCRs are also a risk factor for increasing an individual’s susceptibility to stress, as worse PCRs induced more internalizing problems, and could not help adolescents to effectively cope with stress [59].

Second, when individuals who suffer unavoidable constraints of their fate (flood and COVID-19), deny that there exist constraints of their fate, or do not believe that their efforts could compensate their fate, they may choose more passive coping strategies and negative cognition reappraisal [21,22,60]. They may tend to ignore the potential social resources of their parents; thus, their high PCRs would not help them to actively cope with the strain in disasters. They may prefer to harass others in cyber to try to buffer psychological distress. Further studies are necessary to address these issues.

### 4.3. The Moderating Effects of Parent–Child Relationships

The current study found unexpected but interesting results, suggesting that the moderated effects of PCR were non-significant. Thus, Hypothesis 2 is not supported. This finding could be explained by family system theory: from the perspective of family system theory, there is a dynamic system of interactions and dependencies between family members [61] (pp. 249–272). Although parents have a good relationship with their children, parents’ negative emotional states (e.g., stress, anxiety, worry) in double disasters could affect their children’s emotions or behaviors (i.e., spillover effect) [62,63]. Therefore, adolescents still have higher psychological distress, leading to more cyberbullying behaviors. Previous research supports these explanations by revealing that fathers’ negative mood leads to their children’s greater externalizing problems [64]. Future research could examine these explanations by exploring the roles of parents’ psychological distress and PCRs simultaneously.

## 5. Conclusions

Unlike previous studies that focused on single-level ecological system factors, we explore the antecedent variables of cyberbullying from multiple levels (i.e., individual factors: psychological distress; microsystem factors: parent–child relationships; macro-system factor: cultural beliefs) in the context of double disaster events (floods and the COVID-19 pandemic in China). Our findings demonstrate a significant moderating effect of parent–child relationships and negotiable fate on the relationship between psychological distress and cyberbullying among adolescents experiencing double disasters. 

There are several limitations to the present study: First, we adopted a cross-sectional design, which could not draw causal inferences. Future studies should include longitudinal and experimental studies. Second, the current study used a self-reported method to collect data, and further studies may benefit from combined self-reported, peer/parent-reported data, and physiological methods. Third, it is unclear whether perceived disaster severity affects cyberbullying behaviors [65]. Future studies should consider other factors. Fourth, our study sample was collected from a junior middle school in the middle provinces of China. As the belief in a negotiable fate is spreading in China and other Eastern cultures compared to in Western cultures [21,22], it is necessary to draw inferences from the conclusions of this study for broader cultural contexts. Fifth, according to general strain theory, we fixed psychological distress as an independent variable in the present study’s three-way interaction model analysis. Considering the results of the significant three-way interaction effects in the present study, future studies could explore the potential moderating role of psychological distress by adopting a longitudinal design.

Despite the limitations above, our research has two main theoretical contributions to scientific knowledge: First, this study underlines the value of social-ecological system theory to deepen our understanding of cyberbullying behaviors. Specifically, the findings suggest that the interaction effects of individual factors and microsystems (PCRs) depend on the degree of the macrosystem (culture belief); the latter factor is underestimated in previous cyberbullying research. This study also expands the theoretical scope of the social-ecological system and general strain theory in non-Western cultural contexts. Second, this is the first study to test the direct and moderating effects of psychological distress, PCRs, negotiable fate, and cyberbullying among Chinese adolescents who suffer from a succession of flood and COVID-19 disasters in one month. Although scholars have suggested that COVID-19 may permanently change human life [58], and how to help human beings adapt their (online) life in the COVID-19 era is a duty of researchers [58], empirical studies exploring the effects of multiple disasters on human cyber behavior are limited. Considering that disasters such as floods, earthquakes, and terrorist attacks can still occur simultaneously in the context of the persistence of the COVID-19 pandemic, this research expands the scope of cyber behavior research, helping to inspire future studies examining online social behaviors in the context of double disasters. 

These findings have practical implications for cyberbullying prevention programs. First, our findings underline the value of intervening at the individual-level factors to prevent cyberbullying behaviors in double disasters. Second, according to the results of the moderated moderating model in the current study, schools could develop more targeted interventions when double disasters break down. Specifically, schools in eastern countries may prioritize the selection of students with higher psychological distress, lower belief in negotiable fate, and worse PCRs in double disasters for an intervention, as these students are more likely to perform cyberbullying behaviors. 

## Figures and Tables

**Table 1:** Descriptive statistics and correlations (*N* = 1204).

	**1**	**2**	**3**	**4**	**5**	**6**	**7**
1. Psychological distress	1						
2. PCRs	−0.33 **	1					
3. Negotiable fate	−0.18 **	0.27 **	1				
4. Cyberbullying	0.15 **	−0.14 **	−0.14 *	1			
5. Gender (Male = 1)	0.11 *	−0.10 **	0.01	−0.00	1		
6. Grade	−0.05	0.03	0.04	0.01	−0.03	1	
7. Victimization	0.21 **	−0.18 **	−0.03	0.19 **	0.02	−0.03	1
*M*	16.56	16.25	4.68	1.13	1.48	1.89	0.07
*SD*	18.23	3.38	0.95	0.43	0.50	0.80	0.28

Note. Gender was coded as 1 = male, 2 = female. PCRs = parent-child relationships. The mean of child sex reflects the percentage of male students. * *p* < 0.05; ** *p* < 0.01.

**Table 2:** Model summary of the moderated moderating effect of parent–child relationships and negotiable fate in the associations between psychological distress and cyberbullying.

**Variables**	***β***	***SE***	***t***	***p***	**95% CI**	
Outcomes = Cyberbullying						
Psychological distress	0.03	0.01	2.30	0.02 *	0.01	0.06
PCRs	−0.03	0.01	−2.09	0.04 *	−0.05	−0.001
Negotiable fate	−0.05	0.01	0.19	0.00 **	−0.08	−0.03
Psychological distress × PCRs	0.002	0.01	−3.86	0.85	−0.02	0.02
Psychological distress × Negotiable fate	−0.04	0.01	−2.81	0.01 *	−0.06	−0.01
PCRs × Negotiable fate	0.003	0.01	0.36	0.72	−0.02	0.02
Psychological distress × PCRs × Negotiable fate	−0.02	0.01	−2.18	0.03 *	−0.03	−0.001

Note. PCRs = parent-child relationships. These values are based on unstandardized path coefficients. All parameter estimates and significance tests were based on 5000 bootstrapped samples. Significant effects were determined by both 95% CIs that did not include zero and *ps* < 0.05. * *p* < 0.05; ** *p* < 0.01. Covariates included gender, grade, and victimization.

## Data Availability

The data presented in this study are available upon request from the corresponding author.
